# Compositional Effects on Chemical Ordering, Local Atomic Pressure and Thermal Stability in Truncated Octahedral Pd-Ir-Rh Trimetallic Nanoalloys

**DOI:** 10.3390/nano15241895

**Published:** 2025-12-17

**Authors:** Tuğba Göcen

**Affiliations:** Ahmet Erdogan Vocational School of Health Services, Zonguldak Bülent Ecevit University, 67100 Zonguldak, Turkey; tugba_gocen@beun.edu.tr; Tel.: +90-3722613348

**Keywords:** trimetallic nanoalloys, optimization, DFT, atomic pressure, melting, palladium, iridium, rhodium

## Abstract

This study presents a comprehensive atomistic investigation of the structural, mechanical, and thermal properties of Pd_60_Ir_n_Rh_19−n_ trimetallic nanoclusters adopting a truncated octahedral geometry. The compositional evolution of chemical ordering, local pressure distributions, and melting behavior was systematically analyzed using Gupta potential-based basin-hopping global optimization. The accuracy of the Gupta potential predictions was further validated for all configurations using density functional theory (DFT) calculations. The surface layer consisted solely of Pd atoms and was held constant throughout the study. Meanwhile, Ir and Rh atoms were distributed within the 19-atom core region, allowing a detailed evaluation of how variations in core composition affect the energetic and thermal stability of the clusters. The Pd_60_Ir_6_Rh_13_ configuration exhibits the minimum value of mixing energy, corresponding to the most symmetric and energetically stable atomic arrangement. Local pressure analyses showed that Ir incorporation enhances internal compressive stress and induces tensile relaxation on the Pd surface, achieving an optimal strain balance at n = 6. Melting analyses based on caloric curves and Lindemann indices revealed a non-monotonic dependence of melting temperature on Ir content, with Ir-rich clusters displaying the highest thermal resistance and Rh-rich systems showing reduced stability. These findings clarify how Ir/Rh distribution governs the energetic, mechanical, and thermal response of Pd–Ir–Rh nanoalloys, offering a coherent atomistic framework for understanding their composition-dependent stability.

## 1. Introduction

Metal nanoalloys, composed of two or more different metallic elements, form a distinct class of nanomaterials whose size- and composition-dependent properties differ markedly from both isolated atoms and bulk alloys [1,2,3]. Their high surface-to-volume ratio and large fraction of low-coordinated surface atoms give rise to a dense population of potentially active sites, making nanoalloys highly attractive for applications in catalysis, energy conversion, and chemical transformation [4,5,6]. In such systems, atomic species, geometric structure, and chemical ordering are tightly coupled to physical and chemical behaviour; consequently, most theoretical and computational studies focus on locating the lowest–energy structures and preferred chemical ordering patterns, since these structural characteristics have a substantial impact on catalytic performance, stability [7]. From a theoretical point of view, nanoalloys also provide a rich playground for investigating strain engineering, local pressure distributions, and finite-size thermodynamics in finite systems, which are increasingly relevant for next-generation nanocatalysts [8].

Pd-Ir nanoalloys have been employed as catalysts in a variety of processes, including the preferential oxidation of CO in H_2_-rich streams, selective hydrogenation of nitriles, and hydroconversion reactions, where they exhibit high activity and improved resistance to deactivation compared with monometallic counterparts [9,10,11]. Density functional theory (DFT) and atomistic modelling studies have further demonstrated that Pd tends to segregate to the surface while Ir occupies core or subsurface sites, leading to core–shell or onion-like arrangements that optimise both cohesive energy and catalytic performance [12]. Bimetallic systems involving rhodium, such as Pd-Rh and Rh-rich nanoalloys, also form an important class of catalysts. Rh-based nanostructures are known to be highly active in hydrogenation, NO reduction, and CO oxidation reactions [13,14,15,16]. For Pd–Rh nanoalloys, both experiment and computation have indicated that gas-phase adsorbates can induce reversible changes in chemical ordering and even core–shell inversion, with the distribution of Pd and Rh adapting to the surrounding molecular environment [17,18,19]. Despite this progress on binary systems, significantly less attention has been devoted to trimetallic nanoalloys. Trimetallic nanoalloys offer an additional compositional degree of freedom that can be exploited to decouple and tune structural, electronic, and strain effects beyond what is achievable in bimetallic systems [20,21]. In particular, combining Pd with both Ir and Rh in a single nanocluster opens the possibility of simultaneously optimising hydrogen adsorption, resistance to CO-like poisons, and thermal stability, while engineering specific core–shell or multi-shell arrangements to target desired reaction pathways [1,3,9,12,17,22].

Experimental studies have demonstrated that Pd-based multimetallic nanoparticles containing Ir and Rh can be synthesised using solution-phase reduction routes such as ethylene–glycol–assisted polyol reduction and co-reduction methods, typically yielding particles in the 2–5 nm size range [9,22]. Trimetallic systems incorporating Pd, Ir and Rh have also been successfully produced through chemical reduction in polyol media, confirming that such multimetallic combinations are experimentally accessible [23].

Existing trimetallic modelling work has largely focused on other element combinations and on global energetic trends [24,25]. Strain and local atomic pressure are now recognised as key descriptors for catalyst design in metal nanoalloys. Lattice mismatch between different components generates compressive and tensile strain fields that can shift the d-band centre of surface atoms and thereby modulate the adsorption strength of reactive intermediates [26,27,28,29].

Moreover, local stress and strain are often highly inhomogeneous in finite clusters, with vertices, edges, and facets experiencing different pressure environments; these variations can stabilise or destabilise particular adsorption sites, affect activation barriers, and even drive strain-induced restructuring under reaction conditions [30,31,32]. In this context, mapping how chemical ordering in multicomponent nanoalloys translates into local pressure distributions provides a powerful route to connect atomic scale structure with catalytic performance [30,33,34]. From the structural point of view, truncated octahedral (TO) motifs are among the most commonly observed shapes for face-centred cubic (fcc) metal nanoclusters [3,35]. Experimental transmission electron microscopy (TEM) and scanning TEM studies have reported that Pd-rich and Pd-Ir nanoparticles frequently adopt TO geometries, which are consistent with their fcc crystal structure and minimise surface energy by exposing a combination of {111} and {100} facets [11,22,35].

In this study, Pd_60_Ir_n_Rh_19−n_ nanoclusters were constructed in the (TO) geometry. The TO morphology is an FCC-derived structure that minimizes surface energy through a balanced combination of {111} and {100} facets [36]. Experimental investigations of noble-metal nanoparticles, including the Au_309_ clusters examined by Chen, Li, and Johnston, show that the TO geometry is frequently used as one of the representative models for FCC nanocrystals [37]. First-principles studies of noble-metal nanoalloys further support the use of the TO architecture, as TO clusters have been employed to analyse segregation, reconstruction, and adsorption processes, confirming that this geometry provides a realistic and computationally reliable representation of FCC nanoparticles [38]. Moreover, the TO structure offers a consistent set of symmetry-distinct atomic environments, enabling systematic evaluation of composition-dependent chemical ordering and local stress variations. For these reasons, the TO geometry is adopted in the present work [39].

Trimetallic nanoalloys exhibit synergistic behaviors, including tunable segregation, multi-component strain fields, enhanced stability, and modified catalytic activity, that cannot be achieved in binary systems. Although Pd-Ir and Pd-Rh binary nanoalloys have been studied previously, no work has systematically explored the full Ir/Rh compositional continuum in Pd-based trimetallic clusters. The present work fills this gap by mapping how Ir/Rh substitution reshapes chemical ordering, atomic pressures, and thermal stability of ternary Pd_60_Ir_n_Rh_19−n_ across the entire composition range.

This study presents a systematic theoretical investigation of trimetallic Pd-Ir-Rh nanoalloys in TO geometries. A many-body Gupta interatomic potential parameterised for late transition metals is employed to explore the configuration space of Pd-Ir-Rh clusters through optimisation techniques, enabling the identification of low-energy chemical-ordering patterns across a wide range of compositions. The analysis focuses on high-symmetry TO clusters in the small-size regime, where all three elements can occupy both core and surface sites and where finite-size and strain effects are particularly pronounced. For each composition and low-energy isomer, mixing energies, site-resolved chemical ordering, and local atomic pressures are evaluated to elucidate how size mismatch and elemental distribution give rise to inhomogeneous stress fields. By correlating chemical ordering with local pressure distributions, the study provides microscopic insight into the structural factors that influence the stability of Pd-Ir-Rh nanoalloys, offering a composition-dependent perspective on their energetic and mechanical behavior.

## 2. Materials and Methods

### 2.1. The Optimization Details

In this study, the structural and thermal properties of 79-atom Pd_60_Ir_n_Rh_19−n_ (n = 0–19) trimetallic nanoclusters with TO geometry were investigated through a combination of global optimization and molecular dynamics simulations. To model the interatomic interactions, the many-body Gupta potential was employed, which is derived from the second-moment approximation of tight-binding theory [40,41]. This potential captures both repulsive and attractive interactions, allowing for a reliable description of metallic bonding in complex nanoalloys.

The total potential energy V of the nanocluster system composed of N atoms is expressed as:

(1) $$V=\sum_{i=1}^{N}V_{i}^{r}-V_{i}^{m}$$

where $V_{i}^{r}$ denotes the repulsive pairwise interaction for atom i and $V_{i}^{m}$ represents the attractive many-body term. These components are defined by:

(2) $$V_{i}^{r}=\sum_{j\neq i}^{N}A\left(a,b\right){exp}^{-p\left(a,b\right)\left(\frac{r_{ij}}{r_{0}\left(a,b\right)}-1\right)}\;$$

(3) $$V_{i}^{m}=\sqrt{\sum_{j\neq i}^{N}\zeta^{2}\left(a,b\right)\;{exp}^{-2q\left(a,b\right)\left(\frac{r_{ij}}{r_{0}\left(a,b\right)}-1\right)}\;}\;$$

Here, a and b refer to the atomic species of atom i and j, rᵢⱼ is the distance between atoms i and j, and the parameters A, r_0_, ζ, p, and q are fitted based on cohesive energy, lattice constant, and elastic constants of the corresponding bulk materials at 0 K. The Gupta potential parameters were taken from the article by Cleri and Rosato [42] listed in Table 1. Parameters for heteronuclear interactions were derived using the arithmetic mean (for p, q, and r_0_) and the geometric mean (for A and ζ) of the homonuclear values [43]. This geometric–arithmetic mixing scheme is the conventional and theoretically well-grounded choice for Gupta/TB-SMA potentials and has been widely used in previous studies on transition-metal and noble-metal nanoalloys [3,12,44,45].

Although the Gupta (TB-SMA) potential has been widely and successfully applied to fcc transition-metal nanoalloys, it is important to acknowledge its intrinsic limitations. As a second-moment approximation to a tight-binding Hamiltonian, the model does not explicitly incorporate charge transfer, polarization, or orbital hybridization effects. Consequently, subtle electronic-structure phenomena, such as d-band shifts, ligand-field interactions, and element-specific variations in local density of states, cannot be directly captured. Furthermore, Gupta parameters are derived from bulk cohesive properties (cohesion energy, lattice constant, elastic constants), which means that coordination-dependent electronic rearrangements at low-coordination sites may be represented only approximately. These simplifying assumptions can introduce small deviations in heteronuclear bonding strengths, local pressure distributions, and environment-sensitive energetics, particularly for systems containing elements with markedly different electronegativities or d-electron populations. Despite these limitations, extensive benchmarks demonstrate that Gupta potentials provide a robust and physically meaningful description of structural motifs and segregation patterns for fcc nanoalloys up to approximately 2 nm in size [31,42].

To identify the most energetically favorable chemical ordering configurations of the Pd_60_Ir_n_Rh_19−n_ (0 ≤ n ≤ 19) nanoclusters with a TO geometry, chemical ordering optimization calculations were performed using the Monte Carlo Basin-Hopping algorithm [46,47] within the framework of the Gupta many-body potential. For each composition, at least 2.0 × 10^6^ Monte Carlo steps were executed, during which atomic identities were randomly permuted to explore different Ir/Rh distributions within the 19-atom core. Exchange moves were used during local relaxation stages to ensure efficient sampling of the potential energy surface and to avoid convergence to local minima [30,48].

### 2.2. DFT Re-Optimization

To assess the structural reliability and energetic stability of the lowest-energy configurations obtained from Gupta-based Basin-Hopping global optimization, density functional theory (DFT) relaxations were performed for Pd_60_Ir_n_Rh_19−n_ (n = 0–19) compositions. All DFT calculations were carried out using the plane-wave self-consistent field (PWscf) code implemented in the Quantum ESPRESSO (v7.2) package [49].

The exchange–correlation interactions were treated within the generalized gradient approximation (GGA) using the Perdew–Burke–Ernzerhof (PBE) functional [50,51], which has been widely validated for structural predictions of metallic systems. Scalar-relativistic projector augmented-wave (PAW) type pseudopotentials were used to model electron–ion interactions for Pd, Ir, and Rh atoms. The kinetic energy cutoff for wavefunctions was set to 40 Ry, and 448 Ry was employed for charge density. These cutoff values were found sufficient to ensure total energy convergence across all systems.

To mimic isolated clusters and suppress spurious periodic interactions, each nanoalloy was placed in a cubic simulation box with a side length of 20 Å. Due to the finite nature of the clusters, Brillouin-zone integration was restricted to the Γ-point. Smearing of the electronic states was applied using the Marzari–Vanderbilt smearing scheme with a broadening width of 0.02 Ry. A sufficiently small mixing factor was adopted in the self-consistent field iterations to guarantee convergence.

Geometry optimizations were conducted until the Hellmann–Feynman forces acting on all atoms were reduced below 0.02571 eV/Å, indicating well-relaxed and physically meaningful structures. In addition to geometry optimizations, the total DFT binding energy ${∆E}_{tot}^{DFT}$ of each ternary nanoalloy composition was evaluated using the expression:

(4) $${∆E}_{tot}^{DFT}=E\left({Pd}_{60}{Ir}_{n}{Rh}_{\left(19-n\right)}\right)-60E\left(Pd\right)-nE\left(Ir\right)-\left(19-n\right)E\left(Rh\right)$$

where $E\left({Pd}_{60}{Ir}_{n}{Rh}_{\left(19-n\right)}\right)$ is the total energy of the relaxed cluster, and $E\left(Pd\right),E\left(Ir\right)$ and $E\left(Rh\right)$ are the self-consistent total energies of the corresponding isolated metal atoms in their ground states. This formulation allows for a quantitative comparison of relative binding energies across the composition space, enabling a consistent assessment of structural stability at the DFT level.

### 2.3. Mixing Energy and Stability Investigation

The compositional stability of nanoalloys is commonly evaluated using the mixing energy parameter, which quantifies the energetic deviation arising from the combination of different atomic species in multicomponent systems. This parameter provides direct insight into the thermodynamic favorability of alloy formation. Negative mixing energy values indicate that atomic mixing is thermodynamically favorable, enhancing the stability of the cluster relative to the unmixed binaries. In contrast, positive values suggest a tendency toward demixing and reduced stability. The composition that yields the most negative mixing energy is considered the most stable, as it reflects the strongest synergistic interaction between constituent atoms.

In this work, the mixing energy $E_{mix}$ of Pd_60_Ir_n_Rh_19−n_ (n = 0–19) trimetallic nanoclusters was calculated at the Gupta potential level. The structural model consists of 60 Pd atoms fixed at the surface of a TO, while the 19-atom interior is occupied by varying amounts of Ir and Rh. The mixing energy for each composition was computed according to the following expression [3,52]:

(5) $$E_{mix}\left(Pd_{60}{Ir}_{n}{Rh}_{19-n}\right)=E\left(Pd_{60}{Ir}_{n}{Rh}_{19-n}\right)-\left(\frac{n}{19}\right)E\left(Pd_{60}Ir_{19}\right)-\left(\frac{19-n}{19}\right)E\left({Pd}_{60}{Rh}_{19}\right)$$

where $E\left(Pd_{60}{Ir}_{n}{Rh}_{19-n}\right)$ is the total energy of the trimetallic nanoalloy obtained from Gupta-level structural optimization $E\left(Pd_{60}Ir_{19}\right)$ and $E\left({Pd}_{60}{Rh}_{19}\right)$ are the total energies of the corresponding binary reference systems with pure Ir or Rh cores, respectively. This formulation provides a quantitative measure of the energetic favorability of Ir-Rh mixing within the core, while maintaining a fixed Pd shell. Consequently, it enables a systematic assessment of stability trends across the entire composition range and serves as a reliable guideline for the rational design of Pd-based trimetallic nanocatalysts with optimized thermal stability, catalytic activity, and structural ordering.

### 2.4. Local Atomic Pressure Calculations

In multimetallic nanoalloys composed of elements with different atomic radii and cohesive energies, internal strain plays a decisive role in determining both structural stability and chemical ordering. This effect is particularly important in systems where atomic size mismatch generates heterogeneous stress distributions. In the present study, Pd_60_Ir_n_Rh_19−n_ (n = 0–19) TO clusters exhibit a fixed Pd-rich surface and variable Ir/Rh core compositions, introducing lattice-mismatch–induced strain that influences segregation behavior and thermal stability.

For each composition, the most stable configuration obtained from Basin-Hopping optimization with the Gupta potential was employed as the reference structure for static (0 K) local pressure calculations. Following the definition given by Ferrando et al. [26,48,53], the per-atom stress tensor σ*_i_* is a 3 × 3 matrix defined for each atom *i*, and the isotropic local pressure $P_{i}$ is obtained from its trace as:

(6) $$P_{i}=-\frac{1}{3}Tr\;(\underline{\sigma_{i}})\;$$

Here, *Pi* > 0 corresponds to compressive stress, *Pi* < 0 to tensile stress, and *Pi* = 0 to a stress-free state, as would be expected in an ideal infinite crystal lattice.

### 2.5. Melting Behavior and Thermal Stability Analysis

The thermal response of Pd_60_Ir_n_Rh_19−n_ nanoalloys was systematically investigated using classical molecular dynamics (MD) simulations. The chemically most stable configurations, identified as global minima by the Gupta many-body potential, were employed as initial structures for the simulations. All calculations were performed in the canonical (NVT) ensemble without periodic boundary conditions [37], and all MD simulations were carried out using the DL_POLY_4 version 5.0.0 package [54,55].

The Andersen thermostat was applied with a relaxation time of 0.5 ps, and the equations of motion were integrated using the Velocity Verlet algorithm [56] with a time step of 0.001 ps. Each simulation was conducted for 280,000 steps (corresponding to 280 ps of physical time), including 50 ps of equilibration and 230 ps of data collection at each temperature. The nanoalloys were gradually heated from 0 K to 1800 K in 20 K increments, with independent simulations performed at every temperature point. This finely resolved protocol enabled the detailed characterization of temperature-dependent structural transformations.

To evaluate melting behavior, caloric curves (internal energy vs. temperature) were generated for each composition. The melting point was identified as the temperature at which a distinct change in the slope of the caloric curve appeared, corresponding to a phase transition. In parallel, the Lindemann index was employed as a microscopic criterion for melting, based on atomic displacement fluctuations. It was computed as [45,57,58,59]:

(7) $$\delta =\frac{2}{N\left(N\;-1\right)}\sum_{j<k}\frac{\sqrt{\langle {r_{jk}}^{2}\rangle -{\langle r_{jk}\rangle }^{2}}}{\langle r_{jk}\rangle }$$

where *N* is the total number of atoms, r_jk_ is the distance between atoms *j* and *k*, and the angle brackets denote time-averaged values at a given temperature. According to the conventional criterion, melting occurs when δ reaches the critical threshold of 0.10–0.15. This formulation provides a quantitative measure of atomic disorder. Together, the caloric curve and Lindemann index analyses provide consistent and complementary criteria for identifying the onset of melting.

## 3. Results

### 3.1. Structural Analysis

**Figure 1:**
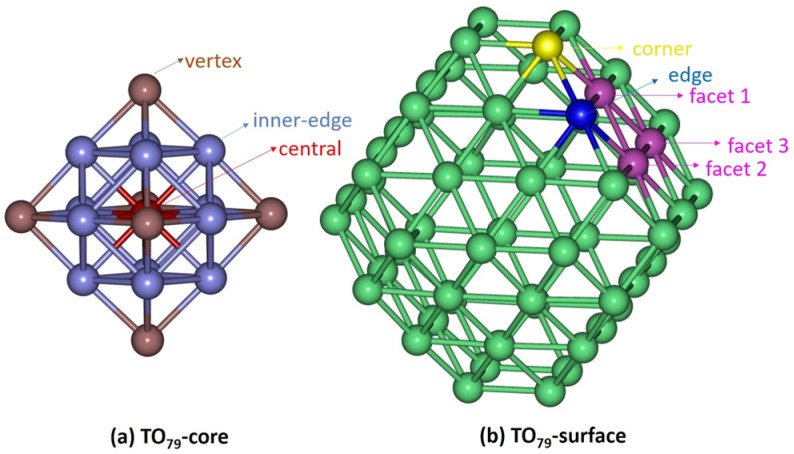
Site labelling for the 79-atom TO nanocluster (19-atom core, 60-atom shell). (**a**) Core sites: central (red), vertex (brown), inner-edge (blue-violet). (**b**) Surface sites: corner (yellow), edge (blue), facet atoms (magenta). In the model, all surface sites are Pd, while Ir/Rh occupy core sites.

The symmetrical positions of the 79-atom TO model, consisting of a 19-atom core region and a 60-atom surface shell, are illustrated in Figure 1. Figure 1a labels the core sites (central: red; vertex: brown; inner-edge: blue-violet), and Figure 1b labels the surface sites (corners: yellow; facets: magenta; edges: blue). This site-resolved framework is used to analyze chemical ordering and local pressure.

In the Pd_60_Ir_n_Rh_19−n_ trimetallic system, the surface shell is entirely composed of Pd atoms, while the core region initially contains only Rh atoms. The core contains one central atom, six vertex atoms, and twelve inner-edge atoms. The surface layer comprises 60 Pd atoms distributed over three crystallographic site classes: (100) corners, (111)–(100) edges, and (111) facets. Specifically, 24 atoms occupy the (100) corners of the six square facets (four per facet), another 24 atoms lie at the centers of the eight (111) facets (three per facet), and the remaining 12 atoms are located along the (111)–(100) edges, i.e., the ridge lines where a (111) facet meets a neighboring (100) facet.

**Figure 2:**
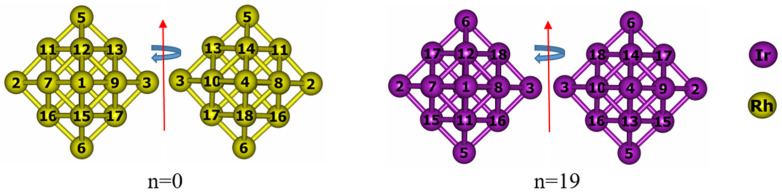
Cross-sectional views of the binary end-members optimized with the Gupta potential: Pd_60_Rh_19_ (n = 0, Rh-only core, (**left**)) and Pd_60_Ir_19_ (n = 19, Ir-only core, (**right**)). Pd surface atoms are omitted for clarity. Arrows indicate the viewing and rotation directions. Color scheme: Rh = yellow, Ir = purple. The numbers shown in the figure correspond to the atom indices of the atoms located in the core region.

**Figure 3:**
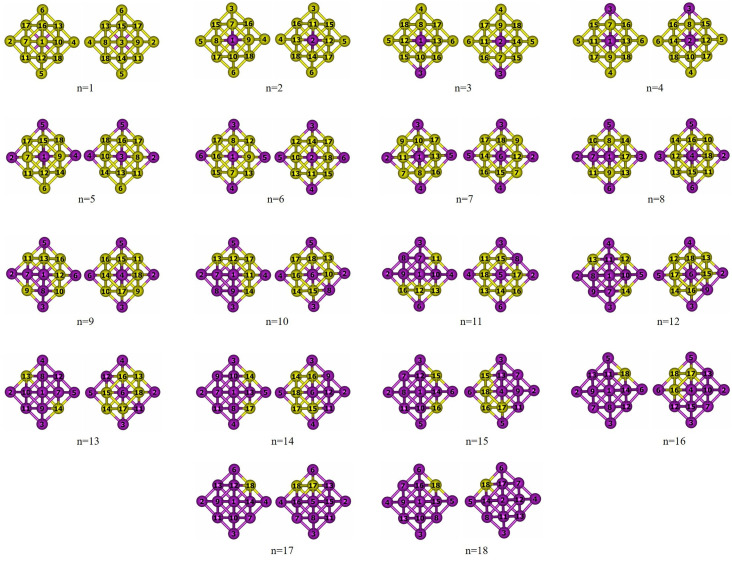
Cross-sectional views of the optimized intermediate compositions Pd_60_Ir_n_Rh_19−n_ (n = 1–18) at the Gupta level. The core contains mixed Rh (yellow) and Ir (purple); Pd surface atoms are omitted for clarity. The numbers shown in the figure correspond to the atom indices of the atoms located in the core region.

The evolution of chemical ordering across the Pd_60_Ir_n_Rh_19−n_ series is summarized in Figure 2 and Figure 3. Figure 2 illustrates the idealized core–shell segregation observed in the two binary end-members, whereas Figure 3 shows the progressive occupation of central, vertex, and inner-edge sites as Ir atoms incrementally replace Rh within the core region.

**Figure 4:**
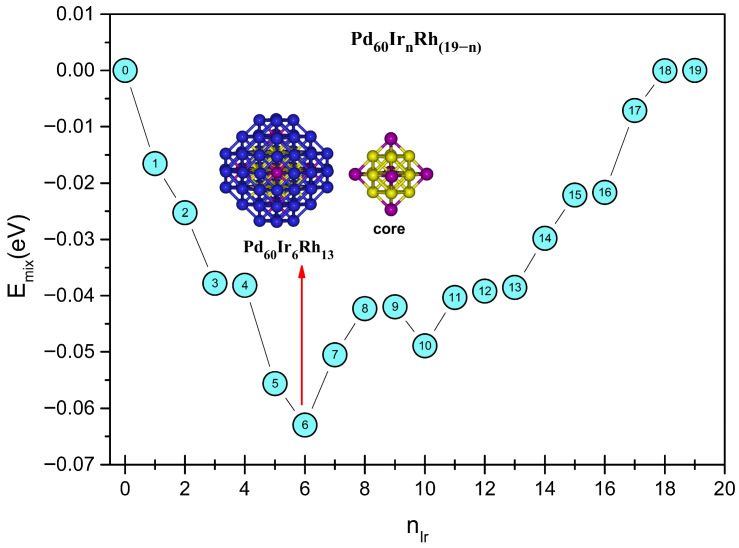
Variation in mixing energy for Pd_60_Ir_n_Rh_19−n_ trimetallic nanoalloys at the Gupta level. Palladium, iridium, and rhodium atoms are represented by blue, purple, and yellow, respectively.

Different chemical ordering configurations were explored by progressively replacing Rh atoms in the core region with Ir atoms. For the binary end-members Pd_60_Rh_19_ and Pd_60_Ir_19_, a perfect core–shell segregation is observed, with the entire core region exclusively composed of Rh or Ir atoms, respectively (Figure 2). When a single Ir atom is introduced (n = 1), it preferentially occupies the central atom site. At n = 2, Ir atoms are located at opposite vertex positions within the core region. Increasing to n = 3, the third Ir atom fills a vertex adjacent to an already occupied vertex, resulting in three Ir atoms situated at vertex positions. For n = 4, however, the additional Ir atom returns to the central site instead of occupying a vertex. In the n = 5 configuration, vertex occupation resumes, and by n = 6, all six vertex sites are fully occupied by Ir atoms, representing the most symmetric distribution among all compositions (Figure 3)**.** This configuration corresponds to the most favorable chemical ordering in the mixing-energy profile, as shown in Figure 4. After the six vertex sites are occupied at n = 6, the seventh Ir atom fills the central site (n = 7). From n = 8 onward, additional Ir atoms begin to occupy inner edge positions. With further addition of Ir atoms, the occupation of inner edge sites progresses systematically, first filling adjacent edges within the same facet and then extending to other facets. This progressive and site-selective filling pattern explains the oscillatory trend observed in the mixing energy curve (Figure 4), as Ir atoms alternate between energetically favorable and less favorable positions.

Cross-sections in Figure 2 and Figure 3 confirm that the Pd surface shell remains intact for all compositions, while only the Ir/Rh distribution within the core changes. This behaviour is consistent with the cohesive and surface-energy hierarchy summarized in Table 2. Ir and Rh, having higher cohesive and surface energies than Pd, are stabilised in high-coordination core sites, whereas Pd, with the lowest surface energy among the three, tends to segregate to the outer shell. Similar segregation trends have been reported previously for Pd-based nanoalloys, where Pd enriches the surface while more strongly bound components occupy the interior [39,60,61,62,63,64].

A well-established segregation pattern in Pd–Ir nanoalloys has been reported in both theoretical and experimental studies. DFT calculations consistently identify Pd-rich shells and Ir-rich cores as the lowest-energy configurations, while Ir-terminated structures are energetically disfavored. This trend is further supported by the energetic ordering of TO Pd–Ir clusters, where Ir@Pd core–shell motifs are found to be the most stable across different cluster sizes [19]. Experimental STEM measurements likewise reveal Pd enrichment at the nanoparticle surface together with Ir localization in the core, in agreement with the segregation behavior examined in the present Pd_60_Ir_n_Rh_19−n_ study [22].

In addition to mixing energy analysis, the first-order energy difference (ΔE) and the second-order energy difference (Δ^2^E) were employed as complementary stability descriptors for Pd-Ir-Rh nanoalloys. The first-order energy difference quantifies the energetic change associated with the sequential substitution of Rh by Ir in the core and is defined as [60,61,62]:

ΔE = E_min_(n + 1) − E_min_(n) (8)

where E_min_(n + 1) and E_min_(n) represent the minimum total energies of the Pd_60_Ir_n_Rh_19−n_ clusters containing n + 1 and n Ir atoms in the core, respectively.

The second-order energy difference provides a more rigorous criterion by comparing a given composition with its two adjacent counterparts and is expressed as [65,66]:

Δ_2_E = E_min_(n + 1) + E_min_(n − 1) − 2E_min_(n) (9)

Here, E_min_(n) denotes the minimum total energy of the cluster containing n Ir atoms, while E_min_(n − 1) and E_min_(n + 1) correspond to the neighboring compositions with one fewer and one additional Ir atom, respectively. A large positive Δ^2^E value indicates that the cluster at composition n is relatively more stable than its adjacent configurations. Taken together, ΔE and Δ^2^E complement the mixing energy analysis by capturing stability fluctuations across successive compositions and provide a comprehensive description of stability trends throughout the entire Pd_60_Ir_n_Rh_19−n_ series.

**Figure 5:**
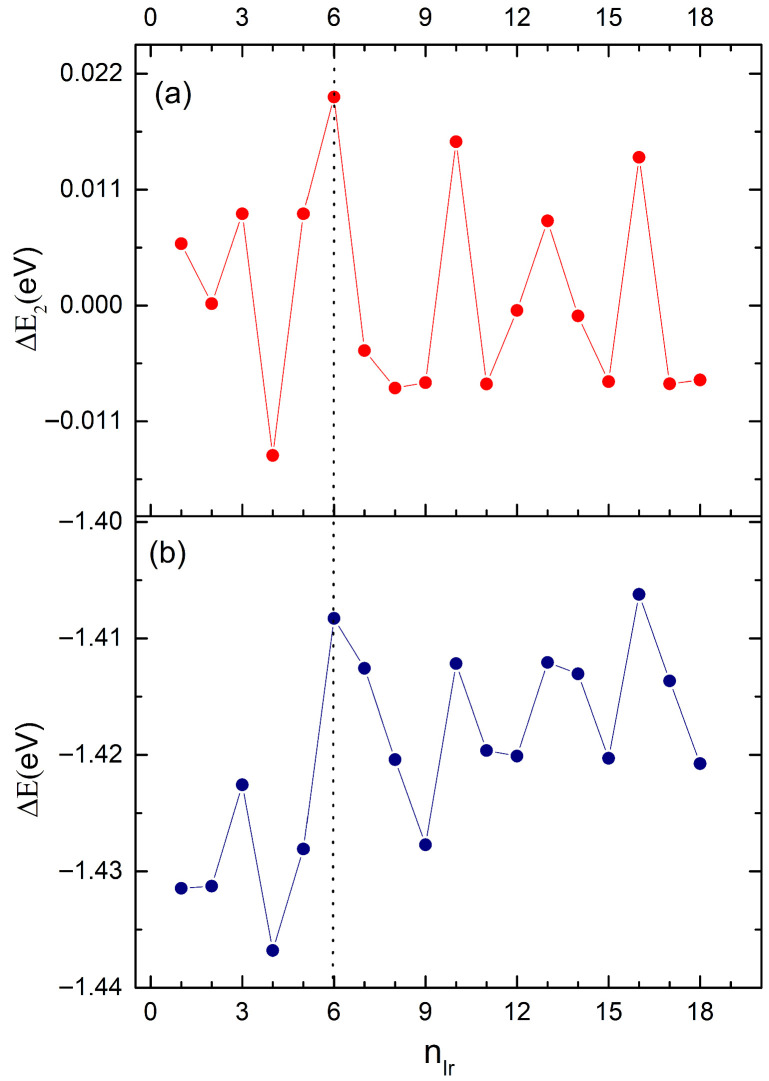
(**a**) Second-order energy differences (Δ_2_E) and (**b**) first-order energy differences (ΔE) of Pd_60_Ir_n_Rh_19−n_ clusters as a function of Ir content (n). A dashed vertical guide line at n = 6 highlights the composition where the most pronounced energetic fluctuations appear.

Energy difference analysis (ΔE and Δ^2^E) provides further insight into the stability of Pd_60_Ir_n_Rh_19−n_ clusters (Figure 5). The first-order energy difference (ΔE) remains negative across all compositions, confirming strong binding, while small fluctuations reflect site-specific occupations (vertex, core, edge). The second-order energy difference (Δ^2^E) shows oscillatory behavior, with positive peaks (notably at n = 6) marking enhanced stability relative to neighboring compositions. The sharp maximum at n = 6 coincides with the global minimum of the mixing energy curve, indicating Pd_60_Ir_6_Rh_13_ as the most stable configuration.

### 3.2. Density Functional Theory (DFT) Calculations

**Figure 6:**
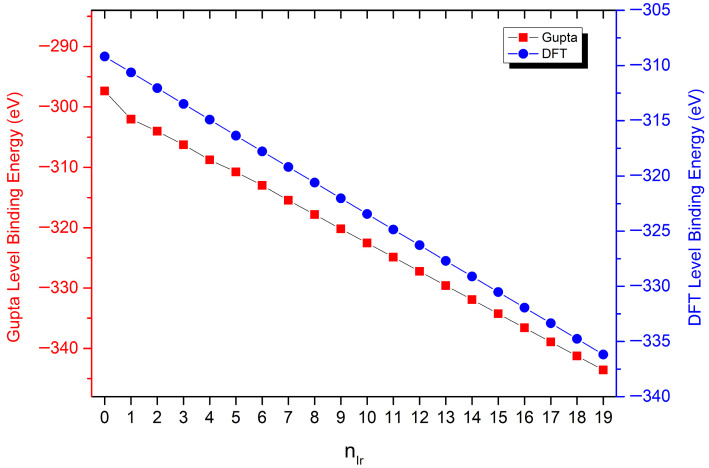
DFT and Gupta binding energies for Pd_60_Ir_n_Rh_19−n_ nanoalloys as a function of Ir content (n).

To gain deeper structural insight into Pd_60_Ir_n_Rh_19−n_ nanoalloys, the Gupta many-body potential was combined with density functional theory (DFT). Chemically ordered minima obtained at the Gupta level were subsequently re-optimized with DFT to assess energetic stability and atomic configurations. The two approaches appear largely consistent: DFT-relaxed structures retain the TO morphology and differ only by small bond-length adjustments, which suggests that Gupta provides generally reliable starting minima for this system. Figure 6 compares the composition-dependent binding energies obtained from Gupta and DFT, where the overall trend is well reproduced (Pearson correlation coefficient R = 0.96), despite a method-specific offset in absolute energy values. Furthermore, the chemical ordering predicted by the Gupta potential remained unchanged after DFT relaxation for all examined compositions, and the structural deviation between Gupta and DFT geometries was very small (RMSD ≈ 0.045 Å), confirming the local stability of the Gupta-derived configurations at the DFT level. These findings confirm that, while the Gupta potential abstracts away detailed electronic-level interactions, it nonetheless captures the correct energetic hierarchy and segregation physics of Pd-Ir-Rh nanoalloys, providing a reliable framework.

### 3.3. Local Pressure Analysis

After establishing the site-specific chemical ordering and stable configurations in Pd_60_Ir_n_Rh_19−n_ clusters, the mechanical response was analyzed by computing local pressures. In this section, P > 0 denotes compressive stress, P < 0 denotes tensile stress, and P ≈ 0 indicates a nearly stress-free state. The calculated values were evaluated with site-resolved histograms that show how compressive and tensile stresses are distributed over atoms occupying distinct structural sites (central, vertex, inner-edge, facet, corner, edge). This representation enables a direct link between chemical ordering, site occupancy, and the mechanical response in the 79-atom TO nanoclusters.

**Figure 7:**
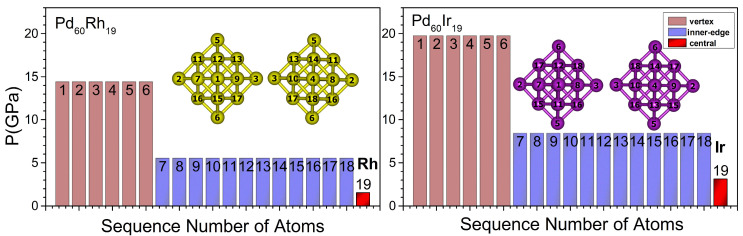
Local atomic pressure profiles for the binary end-member nanoalloys Pd_60_Rh_19_ and Pd_60_Ir_19_. The bars represent the site-specific pressures for atoms occupying vertex, inner-edge, and central positions within the TO geometry. The numbers shown in the figure correspond to the atom indices of the atoms located in the core region.

**Figure 8:**
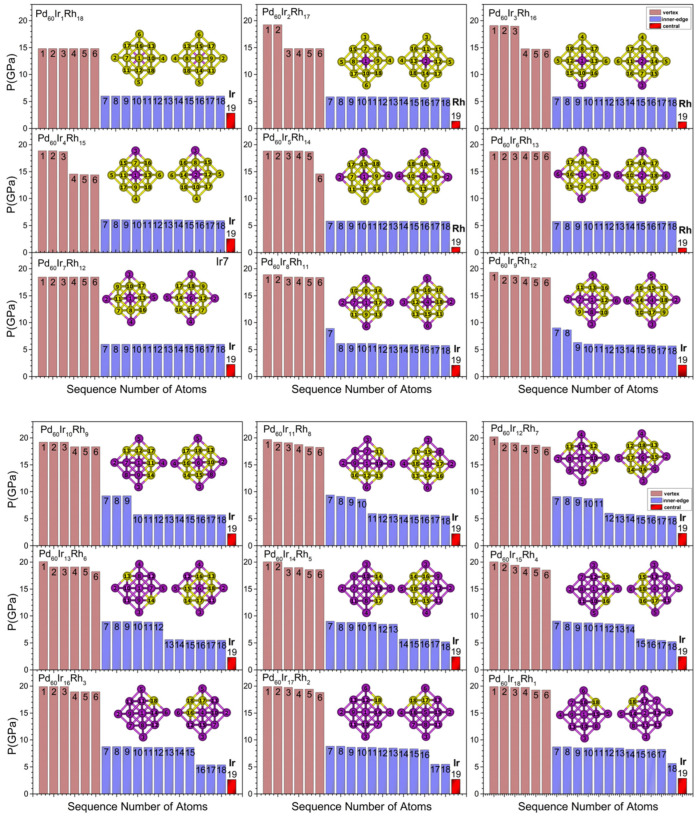
Local pressure profiles at internal occupation sites in Pd_60_Ir_n_Rh_19−n_ (1 ≤ n ≤ 18) nanoalloys. The bars represent the site-specific pressures for atoms occupying vertex, inner-edge, and central positions within the TO geometry. The numbers shown in the figure correspond to the atom indices of the atoms located in the core region.

**Figure 9:**
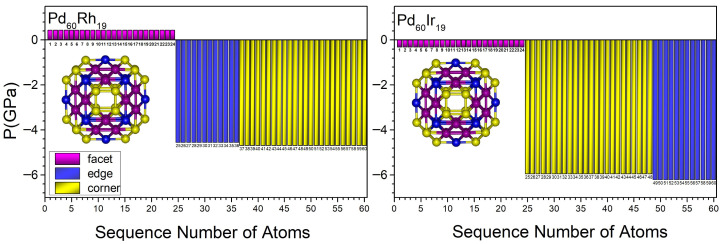
Local atomic pressure profiles for the binary end-member nanoalloys Pd_60_Rh_19_ and Pd_60_Ir_19_ at different surface occupation sites. The bars represent the site-specific pressures for atoms located at facet, edge, and corner positions within the TO surface geometry. The numbers shown in the figure correspond to the atom indices of the atoms located in the surface region.

**Figure 10:**
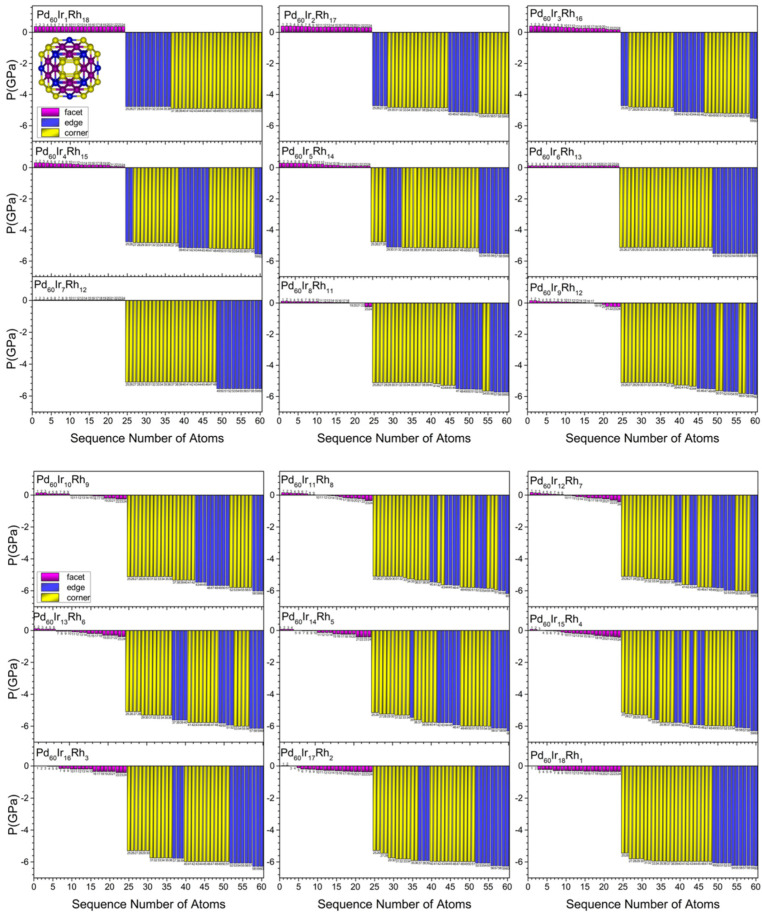
Local atomic pressure profiles at surface occupation sites in Pd_60_Ir_n_Rh_19−n_ nanoalloys (1 ≤ n ≤ 18). The bars represent the site-specific pressures for atoms located at facet, edge, and corner positions within the TO surface geometry as the surface composition evolves with Ir substitution. The numbers shown in the figure correspond to the atom indices of the atoms located in the surface region.

Figure 7, Figure 8, Figure 9 and Figure 10 present the local pressure profiles for the different sites. In the core histograms (Figure 7 and Figure 8), bars are colored by site exactly as in Figure 1a, and the number above each bar denotes the atom index. Figure 7 shows the internal distributions for the binary end-members Pd_60_Rh_19_ and Pd_60_Ir_19_; Figure 8 extends the analysis to intermediate compositions. For the surface, the histograms in Figure 9 and Figure 10 follow Figure 1b surface-site color scheme (facet, corner, edge) and report the corresponding local pressure distributions.

As shown in Figure 7, for the binary end-members Pd_60_Rh_19_ and Pd_60_Ir_19_, the local pressures at the core vertex, inner-edge, and central sites are compressive. The magnitudes of these compressive pressures are lower in Pd_60_Rh_19_ than in Pd_60_Ir_19_. This suggests that Ir strengthens compressive fields in the core and increases mechanical confinement.

Across intermediate compositions (Figure 8), at n = 1, the central atom is Ir and the central compressive pressure increases, accompanied by small rises at neighboring vertex and inner-edge sites. At n = 2, two Ir atoms occupy opposite vertex positions, and these two vertices carry higher compressive pressure than the remaining vertices. At n = 3, a third Ir fills a vertex adjacent to an occupied vertex, placing three vertices under higher compressive stress. At n = 4, the added Ir moves to the central site; the central compressive stress increases modestly while vertex pressures slightly relax. At n = 5, vertex filling resumes, and at n = 6 all six vertices are occupied by Ir, yielding the most symmetric core and coinciding with the global minimum in mixing energy (Pd_60_Ir_6_Rh_13_). At n = 6, all vertex atoms share the same local pressure (18.7 GPa) and all inner-edge atoms share the same local pressure (5.7 GPa); hence, the core pressure map exhibits a uniform distribution. For n > 6, Ir (after the center is Ir again at n = 7) fills inner-edge positions facet-by-facet; both vertex and inner-edge sites then show gradual compressive increases with small composition-sensitive oscillations. In the lowest-energy structures, the central atom is Rh at n = 2, 3, 5, and 6; in these few compositions the central positive (compressive) pressure is lower than when the center is occupied by Ir. From n ≥ 7 onward, the center is always Ir and the central compressive pressure increases monotonically. As a result, inside the core, when Ir occupies a given site, the local positive (compressive) pressure is always higher than when the same site is occupied by Rh. In contrast to the core behavior, the evolution of surface-site pressures is presented in Figure 9 and Figure 10. Figure 9 compares the local pressures at corner, edge, and facet sites for the two binary end-members, highlighting the consistently higher compressive stresses generated when Ir occupies a given surface site instead of Rh. Figure 10 extends this analysis to intermediate compositions (n = 1–18), showing how surface pressures evolve systematically as the Ir content increases.

As shown in Figure 9 and Figure 10, facet atoms remain near *p* ≈ 0 across all compositions, i.e., nearly stress-free: at n = 0 they are slightly compressive (+0.43 GPa) and at n = 19 slightly tensile (−0.33 GPa). The corner and edge atoms are tensile and larger in magnitude than facets. At n = 0 edges are less tensile than corners (edge: −4.56 GPa, corner: −4.70 GPa), whereas at n = 19 the trend reverses (corner: −5.96 GPa, edge: −6.22 GPa). Up to n = 6, the surface tensile levels vary in step with the progressive Ir filling of core vertex sites, indicating strong coupling between core ordering and surface strain. At n = 6, all facet atoms share the same pressure (+0.03 GPa), all corner atoms share the same tensile pressure (−5.13 GPa), and all edge atoms share the same tensile pressure (−5.52 GPa), so the surface pressure map is symmetric at the mixing energy minimum Pd_60_Ir_6_Rh_13_. For n > 6, tensile stress on surface Pd atoms increases steadily with Ir content and approaches a maximum near n = 19. Thus, the Ir/Rh arrangement in the core strongly correlates with the tensile fields on the Pd surface.

**Figure 11:**
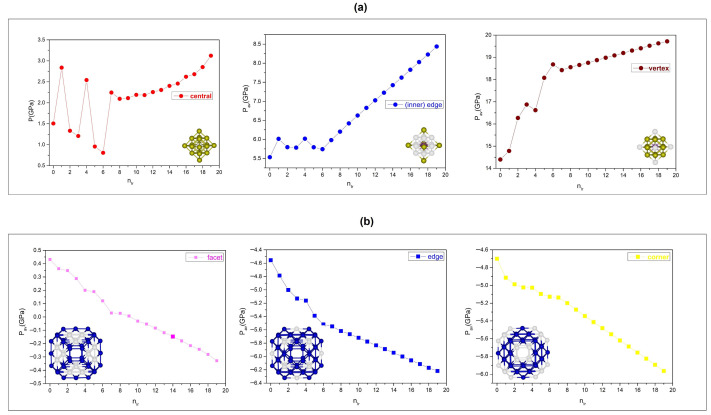
Average local pressures for atoms occupying (**a**) core sites (central, inner-edge, vertex) and (**b**) surface sites (facet, edge, corner) across the Pd_60_Ir_n_Rh_19−n_ composition range. Each panel shows the composition-dependent average pressure for a specific structural site type, illustrating how local environments respond to progressive Ir substitution.

Figure 11 summarizes the average local pressures for the core (central, vertex, inner-edge) and surface (facet, corner, edge) regions. Across all compositions, vertex and inner–edge sites remain under positive (compressive) isotropic local pressure, increasing from Rh-rich to Ir-rich cores. Among core sites, vertex atoms consistently exhibit the highest compressive local pressures, followed by inner-edge atoms, in line with their averages in Figure 11. The compressive pressures of the vertex and inner-edge atoms initially fluctuate in a wavelike manner up to n = 6, and then increase monotonically for n ≥ 6. Initially at n = 0, the average pressure of vertex atoms is about 14.4 GPa and it reaches about 19.7 GPa at n = 19. The average pressure of inner-edge atoms is lower but follows a similar increasing trend, rising from about 5.5 GPa at n = 0 to about 8.43 GPa at n = 19. The single central atom remains under compressive stress at all compositions, and its pressure varies with Ir to Rh substitution at the central site. On the surface, the average pressures at corner and edge sites are consistently negative, which reflects persistent tensile stress. Facet atoms fluctuate around *p* ≈ 0 and act as a strain-balancing layer between the compressive core and the tensile outer region.

In summary, the pressure distributions show a clear link between chemical ordering and mechanical response. Compressive stress dominates in the core, especially at vertex sites. Tensile stress prevails on the surface, mainly at corner and edge sites. The homogeneous stress pattern at n = 6 matches the minimum in mixing energy and supports Pd_60_Ir_6_Rh_13_ as the most stable configuration in both energetic and mechanical terms.

### 3.4. Melting Behavior Analysis

The melting behavior of Pd_60_Ir_n_Rh_19−n_ trimetallic nanoclusters was systematically investigated through caloric curve and Lindemann index analyses. Independent canonical (NVT) simulations were performed for each composition, and the melting points were determined based on the onset of structural transitions. A distinct increase in the Lindemann index coinciding with a slope change in the caloric curve was identified as the melting point.

**Figure 12:**
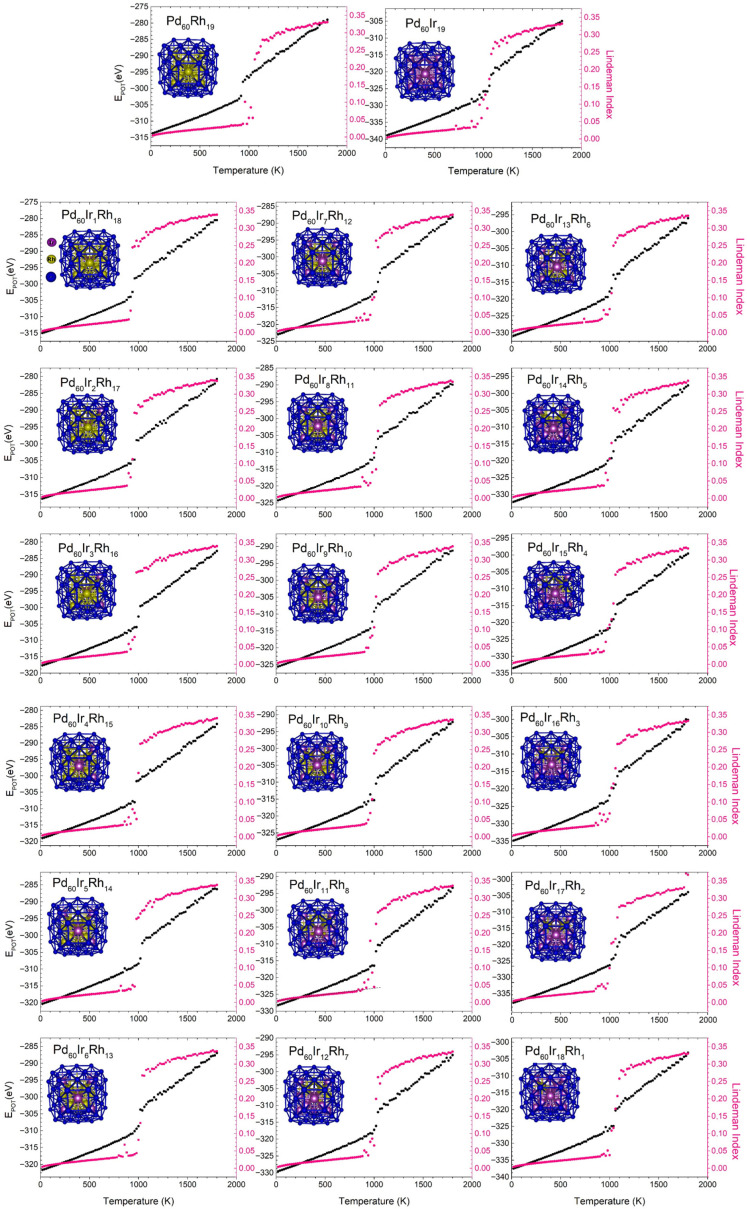
Representative caloric curves (black) and Lindemann index profiles (pink) for the Pd_60_Ir_n_Rh_19−n_ nanoalloys across the full composition range. Each panel shows the temperature-dependent evolution of potential energy and atomic fluctuations, together with the corresponding nanocluster structure for reference.

Figure 12 illustrates the representative caloric curves and corresponding Lindemann index variations for all Pd_60_Ir_n_Rh_19−n_ compositions. Within the same temperature range, the Lindemann index exhibited a clear jump, confirming the melting transition. These two independent indicators provided consistent and complementary evidence of phase transformation.

To establish a more objective criterion, the melting temperature (T_m_) was evaluated by determining the lower (T_1_) and upper (T_2_) bounds of the transition region in the caloric curve and taking T_m_ = (T_1_ + T_2_)/2 (±10 K). This procedure is equivalent to locating the intersection between the low-temperature and high-temperature linear portions of the caloric curve [62].

**Figure 13:**
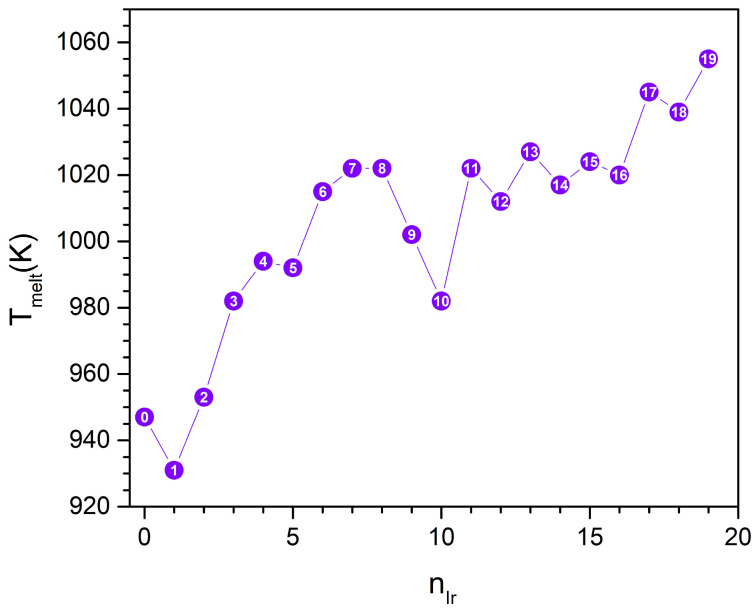
Variation of melting temperature (T_melt_) as a function of Ir composition for Pd_60_Ir_n_Rh_19−n_ clusters. The numbers shown on the purple circles correspond to the number of Ir atoms.

The dependence of the melting temperature on Ir composition is summarized in Figure 13.

Figure 13 presents the variation of melting temperature (T_melt_) as a function of Ir content for Pd_60_Ir_n_Rh_19−n_ nanoclusters, showing a clear non-monotonic trend. Non-monotonic melting trends similar to those observed here have been widely reported in trimetallic nanoalloys, including Cu–Ag–Au, Cu–Au–Pt and Pd–Rh–Pt systems, where the interplay of chemical ordering and strain generates oscillatory thermal stability [67,68,69]. The melting temperatures predicted in this study (933–1058 K) lie within the typical range for 1–3 nm noble-metal nanoparticles and therefore represent physically realistic values [70,71].

In the Rh-rich region (n = 0–1), the melting points are relatively low (933–946 K), indicating that the initial core configuration is more susceptible to thermal disorder. Upon gradual Ir incorporation (n = 2–5), T_melt_ increases progressively (956–986 K), reflecting enhanced structural rigidity as Ir atoms replace Rh at preferred core sites. A pronounced rise in melting temperature is observed for n = 6–8, where T_m_ exceeds 1018 K. This composition range corresponds to the vertex-filled, highly symmetric core configuration, which coincides with the most favorable mixing energy and homogeneous pressure distributions. These observations are consistent with the idea that a symmetric and uniformly strained core can suppress early structural rearrangements. For n = 9–10, the melting temperature decreases slightly (989–1002 K), marking a local instability region. In this regime, Ir atoms begin to occupy inner edge sites, introducing heterogeneous local strain and stress anisotropy within the core. This nonuniformity promotes localized structural relaxation, resulting in a transient decrease in T_melt_ [69]. Beyond n ≥ 11, the melting temperature increases again, reaching its maximum value of 1058 K at n = 19. As Ir content becomes dominant, the core structure evolves toward a more ordered configuration, reducing internal heterogeneity and improving resistance to thermal fluctuations.

In summary, the melting behavior of Pd–Ir–Rh nanoalloys is governed by the combined effects of chemical ordering and local pressure, with thermal stability differing from energetic stability across compositions.

## 4. Conclusions

In this study, a comprehensive investigation of the chemical ordering, local atomic pressures, and thermal behavior of 79-atom TO Pd_60_Ir_n_Rh_19−n_ nanoclusters was conducted. A combined computational framework, based on Basin-Hopping global optimization with Gupta many-body interactions, supported by local structural relaxations and finite-temperature molecular dynamics simulations, was employed to identify the lowest-energy configurations and to characterize their structural evolution across the full Ir/Rh composition range. The comparison of binding-energy trends at the Gupta and DFT levels showed a consistent variation, further validating the reliability of the Gupta potential for describing chemical ordering in this system.

The chemical-ordering analysis revealed pronounced core–shell segregation in the binary end-members (Pd_60_Rh_19_ and Pd_60_Ir_19_), whereas intermediate compositions exhibited composition-dependent redistribution of Ir and Rh atoms among the core sites, while the Pd surface layer remained preserved for all structures. Across the compositional space, a clear correlation between atomic configuration and the resulting strain landscape was established. Local-pressure analyses showed that core atoms are predominantly under compressive stress, while low-coordination surface atoms experience tensile stress, with the (111) facets remaining nearly stress-free and contributing to overall elastic balance. Ir incorporation was found to systematically reshape the internal stress fields: depending on composition, Ir occupies central, vertex, and inner-edge sites within the core, which strengthens the compressive stability of the inner region and partially relaxes tensile stress on the Pd surface. The composition Pd_60_Ir_6_Rh_13_, characterized by symmetric Ir occupation across vertex positions and a homogeneous pressure distribution, exhibited the most balanced core–surface stress partition, consistent with its lowest mixing energy, indicating strong thermodynamic (ground-state) stability.

Melting analyses demonstrated that thermal resistance is strongly influenced by the Ir/Rh distribution within the core. Increasing Ir content enhanced structural coherence and increased melting temperatures, whereas Rh-rich clusters exhibited comparatively reduced thermal stability, showing that thermal stability and thermodynamic (mixing-energy–based) stability peak at different compositions. From a catalyst-design perspective, this distinction highlights that thermodynamic stability governs preferred chemical ordering and strain distribution at working temperatures, while thermal stability is relevant for preventing sintering or structural degradation under high-temperature conditions. Accordingly, Pd_60_Ir_6_Rh_13_ is identified as the most favorable composition in terms of low-temperature energetic preference, whereas Ir-rich compositions such as n = 19 provide superior resistance to melting at elevated temperatures.

Overall, the results identify composition and core-site occupation as key factors governing the mechanical and thermal resilience of Pd–Ir–Rh nanoalloys, offering atomistic insight into the structural principles underlying their composition-dependent stability.

## Figures and Tables

**Table 1:** The Gupta potential parameters for Pd-Ir-Rh trimetallic clusters.

Interaction	A (eV)	ζ (eV)	p	q	r_0_ (Å)
Ir-Ir	0.1156	2.2890	16.9800	2.6910	2.7146
Ir-Rh	0.0853	1.9493	17.7150	2.2790	2.7018
Ir-Pd	0.1421	1.9831	13.9235	3.2165	2.7316
Rh-Rh	0.0629	1.8670	18.4500	1.8670	2.6891
Rh-Pd	0.1048	1.6888	14.6585	2.8045	2.7188
Pd-Pd	0.1746	1.7180	10.8670	3.7420	2.7485

**Table 2:** The cohesive energy (Ecoh), average surface energy (Esurf) and atomic radius (r) of Ir, Rh and Pd atoms [39,56,57].

Atom	E_coh_ (eV)	Esurf (meVÅ^−2^)	r (Å)
Ir	6.94	187.3	1.36
Rh	5.75	168.5	1.35
Pd	3.89	131.0	1.38

## Data Availability

The data presented in this study are available within the article. Any additional data related to this work are available from the corresponding author upon reasonable request.

## Abbreviations

The following abbreviations are used in this manuscript:

TO	Truncated octahedron
fcc	Face-centered cubic
DFT	Density functional theory
MD	Molecular dynamics
NVT	Canonical (constant-NVT) ensemble
GGA	Generalized gradient approximation
PBE	Perdew–Burke–Ernzerhof (GGA functional)
PAW	Projector augmented-wave
PWscf	Plane-wave self-consistent field (Quantum ESPRESSO module)
BH	Basin-hopping
QE	Quantum ESPRESSO
HER	Hydrogen evolution reaction
